# Lifestyle and psychosocial predictors of health resilience among Indonesian disaster preparedness cadres (TAGANA): a machine learning approach

**DOI:** 10.3389/fpubh.2025.1673734

**Published:** 2025-11-05

**Authors:** Mu’man Nuryana, Istiana Hermawati, Sugiyanto Sugiyanto, Asmadi Adnan, Dayat Hidayat, Togiaratua Nainggolan, Setyo Sumarno, Ruaida Murni, Chatarina Rusmiyati, Achmadi Jayaputra, Suryani Suryani, Sri Setyati, Andjar Prasetyo, Alhadi Saputra, Hadi Supratikta

**Affiliations:** ^1^Research and Innovation Agency of the Republic of Indonesia, Research Center for Public Policy, Jakarta, Indonesia; ^2^APMD Village Community Development College, Yogyakarta, Indonesia; ^3^Research Center for Village Social Welfare and Connectivity of the National Innovation Research Agency, Jakarta, Indonesia; ^4^Regional Research and Innovation Agency of South Kalimantan Province, Banjarbaru, South Kalimantan, Indonesia; ^5^Regional Development Planning, Research and Innovation Agency, Magelang City, Central Java, Indonesia; ^6^National Research and Innovation Agency, Jakarta, Indonesia

**Keywords:** disaster preparedness cadets, TAGANA, health resilience, life risk, health expectancy, lifestyle, psychological resilience, volunteer health

## Abstract

This study investigates the determinants of health resilience among TAGANA volunteers in Indonesia using Decision Tree Regression (DTR) and Random Forest Regression (RFR). Data from 200 respondents show that life risk is the most dominant negative factor, while hope healthy acts as a strong protective factor; other variables such as disease history, dynamic experience, and physical-mental balance contribute moderately. RFR outperformed DTR in stability, confirming the robustness of the findings. The results emphasize the need for risk mitigation, psychosocial support, and lifestyle-based interventions, while international best practices provide models for strengthening volunteer resilience. This study contributes evidence-based insights for policy and training frameworks to improve TAGANA’s long-term sustainability in disaster response.

## 1 Introduction

TAGANA play a strategic role in disaster management in Indonesia, a country geographically located in the Pacific Ring of Fire and highly vulnerable to natural disasters and the impacts of global climate change (1, 2). Despite having a vital role, TAGANA’s physical and mental well-being is often overlooked, in contrast to developed countries that consider disaster responders as a systematically protected national asset (3). In fact, a healthy lifestyle and mental readiness have proven to be important factors in maintaining the long-term health resilience of disaster volunteers (4). Conventional statistical methods, while useful, often assume linear relationships and struggle to capture complex, interacting lifestyle and psychosocial variables that characterize disaster volunteer experiences. This limitation necessitates the adoption of more flexible approaches capable of modeling non-linearities and high-dimensional data.

Exposure to trauma, psychological stress, and sub-ideal working conditions, such as lack of rest and lack of access to nutrition, can decrease work effectiveness and increase the risk of disorders such as PTSD, anxiety, and emotional exhaustion (5–7). Interventions such as the Disaster Worker Resiliency Training Program (DWRT) and Psychological First Aid training have been shown to be effective in reducing stress, PTSD, and improving the psychological support competencies of volunteers (8, 9). In addition, a positive mindset toward health (hope healthy) also contributes to adaptability and resilience in stressful conditions (10).

Recent studies show that psychosocial risk factors can affect public health and the well-being of vulnerable populations (11, 12). In the context of volunteers, anxiety, stress, and emotional-cognitive factors play an important role in well-being, so psychological support and therapeutic interventions are needed to prevent fatigue (13–15). In addition, modern psychosocial interventions have been proven to be effective in improving the quality of life and can be a model of long-term support for disaster volunteers (16). Chronic high-risk exposure can lead to cardiometabolic disorders and decreased cognitive function if not balanced with a strong support system (17–19). In contrast, dynamic experiences in disaster situations and social support from fellow volunteers have been shown to improve decision-making skills and mental resilience (20, 21).

This study focuses on the unique context of TAGANA volunteers in Indonesia that has not been widely explored in the global literature, particularly from the perspective of health resilience prediction. Using a machine learning approach, this study aims to identify the dominant factors that affect TAGANA’s health resilience by analyzing lifestyle variables, psychosocial support, disaster experiences, and health history. From a theoretical perspective, resilience is shaped by the dynamic interaction of risk exposure, psychosocial resources, and adaptive behaviors within disaster contexts. Machine learning approaches such as Decision Tree Regression (DTR) and Random Forest Regression (RFR) align with this framework by identifying hidden patterns and ranking the relative importance of predictors, thereby bridging health resilience theory with practical disaster preparedness strategies. For this reason, this study proposes the following hypothesis: *H_1_*: A healthy lifestyle contributes positively to TAGANA’s health resilience; *H_2_*: Hope healthy is positively associated with health resilience; *H_3_*: Exposure to life risks negatively impacts TAGANA’s health resilience; *H_4_*: Physical and mental balance is positively related to health resilience; *H_5_*: A history of chronic illness or previous adverse health conditions negatively impacts health resilience; and *H_6_*: Dynamic experience in disaster situations improves health resilience.

## 2 Method

This study uses a quantitative approach to identify factors that affect the health resilience of TAGANA volunteers. Primary data were collected through structured questionnaires and semi-structured interviews with 200 purposively selected active respondents, with criteria of at least 2 years of experience, age 20–50 years, and willingness to participate. The sample size of 200 respondents was determined based on both methodological precedent in disaster volunteer studies with comparable populations (22, 23) and *a priori* power analysis for multiple regression models (power = 0.80, *α* = 0.05, medium effect size f^2^ = 0.15), which indicated a minimum of 138 participants. Thus, 200 respondents provided sufficient power while accounting for potential attrition or exclusion. The research has obtained ethical approval from BRIN (No. B-1602/III.12.2/FR.01.02/7/2023), and all participants signed a written agreement after receiving a full explanation. Respondents were recruited through coordination with local TAGANA coordinators, and participation was voluntary with informed consent obtained after a clear explanation of study procedures and withdrawal rights. While purposive sampling ensured that only experienced members were included, this approach may introduce selection bias and limit generalizability beyond the defined study population. Data were collected using a Likert scale of 1–5 based on variables developed from previous studies and adjusted to the context of TAGANA, with the assurance of anonymity and confidentiality of participant data. Prior to analysis, the dataset was checked for completeness. Cases with more than 10% missing responses were excluded, while items with ≤5% missing were imputed using mean substitution. Outliers outside the valid range (Likert 1–5) were removed, and all variable indices were standardized using z-scores to ensure comparability across features. For example, if the variable “hope healthy” was measured using 4 items rated on a 1–5 Likert scale, a respondent who scored 4, 5, 3, and 4 would have an index of (4 + 5 + 3 + 4)/4 = 4.0. This standardization allowed comparisons across variables with different item counts. To ensure consistency and comparability across respondents, raw scores were converted into standardized indices using the following formula:


(1) $$I_{i}=\frac{\sum_{j=1}^{n}X_{\mathrm{ij}}}{n}$$

Where: *Ii* is the index score for the *i_-th_* variable; *Xij* represents the score given by the *j_-th_* respondent for the *i_-th_* variable; and *n* is n is the number of indicators (questions) used to measure that variable. Below is a detailed explanation of each variable, including its conceptual definition and how it was quantified. This study examined six key variables influencing the health resilience of TAGANA volunteers. Hope_healthy refers to an individual’s optimism about their future health, measured through self-assessed confidence in managing stress and maintaining a healthy lifestyle (scale 1–5). Healthy_endurance captures the ability to sustain physical and mental wellbeing under pressure, assessed through perceptions of stamina and energy during prolonged or intense situations. Life_risk reflects the perceived vulnerability to physical or psychological harm during disaster response, including concerns about illness or injury while in the field. Physical_mental represents the overall state of physical and mental health, based on indicators like sleep quality, emotional balance, and coping mechanisms. Disease_healthy measures awareness of health risks associated with poor lifestyle habits, such as the link between inadequate sleep and heart disease. Lastly, experience_dynamic captures the impact of repeated disaster exposure on health behavior, assessed by the frequency and intensity of involvement in emergency operations. Each variable was rated on a 1–5 Likert scale. The Likert items were adapted from validated instruments in resilience and health psychology research, then reviewed and refined through expert consultation with BRIN psychologists. Content validity was confirmed by expert judgment, while pilot testing with 30 TAGANA volunteers ensured clarity, cultural appropriateness, and internal consistency (Cronbach’s *α* for the six variables ranged between 0.78 and 0.86). The six variables were selected based on their theoretical importance in resilience and disaster health research, supported by previous studies, and validated through expert consultation with BRIN psychologists to ensure contextual relevance for TAGANA volunteers.

After standardizing the data into indices, the study employed DTR and RFR models for predictive analysis. These machine learning techniques were selected for their ability to model non-linear relationships and handle interactions among variables effectively. The DTR model partitions the dataset into segments (nodes) based on feature values to predict the outcome variable. The prediction at each terminal node is calculated as the average of the target variable within that node:


(2) $$\hat{Y}=\frac{1}{N_{j}}\sum_{i\in R_{j}}y_{i}$$

Where: $\hat{Y}$ is the predicted value of health resilience; Nj is the number of samples in the j-th node; and yi is the actual value of the target variable for sample i in node j. Model performance was evaluated using Mean Squared Error (MSE) and pruning techniques to prevent overfitting. To improve prediction accuracy and reduce variance, the RFR model was also applied. It aggregates predictions from multiple decision trees trained on random subsets of the data:


(3) $$\hat{Y}=\frac{1}{B}\sum_{b=1}^{B}T_{b}X$$

Where: *B* is the total number of trees in the forest; and *TbX* is the prediction made by the b -th tree for input vector X. Model validation was performed using the Out-of-Bag (OOB) Error, which estimates generalization error by testing each tree on data not used during its training. In addition to OOB error, a 10-fold cross-validation was carried out to strengthen model evaluation. This allowed assessment of stability and robustness across different subsets of the data, with performance metrics including MSE and R^2^ reported for each fold. All analyses were conducted using JASP software, chosen for its user-friendly interface and robust statistical capabilities. By combining index-based standardization with machine learning regression techniques, this study provides a comprehensive and data-driven perspective on the determinants of health resilience among TAGANA volunteers. The use of both DTR and RFR allows for capturing both linear and complex patterns in the data, offering deeper insights into how lifestyle, perception, experience, and health history interact to shape resilience in disaster responders.

## 3 Results

The sample consisted of respondents aged 19–70 years, the majority in the productive range (20–50 years), dominated by men (60%), most Muslims (95%), married (70%), with high school educated (60%), and moderate-income (IDR2–5 million). The average family dependents are 2–4 people, higher at the age of >50 years. Occupations vary, with employees (17%), housewives (13%), and self-employed (12%) being the most numerous. Most have been members of TAGANA >10 years (41%) with the main motivation of helping others (46%) and calling of the heart (27%). Humanitarian values (49%) and inner satisfaction (8%) drive sustainability. Education and marital status affect income and family structure, while age correlates with the number of dependents. Decision Tree Regression (DTR) and Random Forest Regression (RFR) analyses showed that the main factors affecting TAGANA’s health resilience were life risk and hope healthy, which were validated through bootstrap resampling and permutation tests to ensure their significance and reliability.

To strengthen statistical claims, 95% confidence intervals and effect sizes were calculated. H2 and H3 showed the strongest effects with narrow confidence intervals, H5 and H6 moderate but significant effects, while H4 remained marginal with a small effect size. This indicates that while central hypotheses are strongly supported, the nuanced role of balance requires cautious interpretation. Beyond statistical significance, variable importance rankings from RFR highlight life risk as the dominant factor decreasing resilience and hope healthy as a key protective factor. These findings suggest the need for occupational safety, psychosocial support, and training programs that strengthen optimism about future health. Other variables such as disease history and physical–mental balance, while less dominant, still indicate the relevance of preventive health education and routine health monitoring for long-term resilience. Taken together, the interpretability measures from RFR allow translation of statistical outputs into actionable health policy. To maximize evidence-based impact, disaster management agencies should focus on reducing life risks through occupational safety and psychological support, and enhance hope and health via training programs. Additionally, they should recognize the supportive role of physical–mental balance and disease prevention. In this study (Table 1), MDA was chosen as the main measure of variable importance because it is more reliable than Mean Decrease Impurity (MDI). DTR analysis confirmed life risk (51.69%) and hope healthy (32.43%) as the strongest predictors of resilience, while disease history (7.44%), dynamic experience (6.02%), and physical–mental balance (2.41%) contributed less. These results are reinforced by the international literature that confirms that life risks, including threats to physical and mental health, significantly affect long-term immunity (24). Meanwhile, hope healthy has been proven to be effective as a buffer for the negative impact of risk, improving quality of life and healthy behaviors (25, 26). Although the contribution of other factors is lower, a balance of physical activity is still important because excessive intensity can actually have a maladaptive impact (27).

**Table 1:** Variable importance levels in DTR and RFR models, results, hypothesis testing and statistical significance.

Variable importance levels in DTR and RFR models				
Variable	Relative importance (%)	Mean decrease in accuracy (MDI)	95% confidence interval	*p*-value
Life risk	51.69	0.027	[48.2, 55.1%]	<0.001
Hope healthy	32.43	0.022	[29.1, 35.7%]	<0.001
Disease healthy	7.44	0.02	[6.2, 8.7%]	0.002
Experience dynamic	6.02	0.011	[5.1, 6.9%]	0.004
Physical mental	2.41	0.009	[1.8, 3.0%]	0.023
The value of the confidence interval indicates the estimated accuracy of the variable’s contribution, while the *p*-value confirms the statistical significance.				

Comparison of DTR and RFR model results				
Type	Test MSE	Dominant factors	OOB error	Interpretation
DTR	9,068 × 10^−4^	Life Risk (51.69%), hope healthy (32.43%)	–	Easy to interpret, but prone to overfitting
RFR	0.002	Life Risk (0.027 MDI), experience dynamic (0.022 MDI)	0.001	More stable due to reduced overfitting

Hypothesis testing and statistical significance				
Hypothesis	Statement	Result	*p*-value	Conclusion
*H_1_*	A healthy lifestyle contributes positively to TAGANA’s health resilience	Accepted	0.002	Significant positive effect
*H_2_*	Hope healthy has a positive relationship with health resilience	Accepted	<0.001	Strongly supported
*H_3_*	Life risk negatively affects TAGANA’s health resilience	Accepted	<0.001	Highly significant negative impact
*H_4_*	Physical and mental balance contributes positively to health resilience	Rejected	0.023	Marginal significance, small effect
*H_5_*	Health history negatively influences health resilience	Accepted	0.002	Moderate negative impact
*H_6_*	Dynamic experience enhances health resilience	Accepted	0.004	Statistically significant

Source: Analysis results, 2025.

The RFR model (Table 1) shows more stable accuracy than DTR with MSE Validation = 6.041 × 10^−4^, Test MSE = 0.002, and OOB Error = 0.001. Both models consistently show that life risk decreases resilience, while hope healthy protects it. RFR additionally highlights the role of dynamic experience (MDI = 0.022), suggesting that field exposure strengthens resilience through adaptation and preparedness (28). High-risk exposure can have long-term impacts such as PTSD and metabolic disorders (17), while expectations of health accelerate recovery through post-traumatic growth (29). DTRs are more explicit in identifying key factors but prone to overfitting, while RFRs are more accurate and stable, although interpretation is more complex.

The results of the analysis of the DTR and RFR models support most of the research hypotheses. H1, H2, and H3 were supported, confirming the dominant influence of life risk and the protective role of hope healthy. H4 showed only a marginal contribution from physical–mental balance (2.41% in DTR; impurity = 0.011 in RFR), indicating limited but non-negligible relevance. H4’s marginal effect (*p* = 0.023; Cohen’s f^2^ = 0.06) may reflect self-report bias or the moderating role of stronger predictors such as life risk and hope healthy. This factor warrants investigation as a long-term predictor in larger or longitudinal studies. H5 and H6 were supported, with disease history showing a moderate negative impact (7.44%) and dynamic experience contributing more strongly in RFR (MDA = 0.022). These findings highlight the need for risk mitigation, preventive health education, and strengthening field experience among TAGANA volunteers.

**Figure 1:**
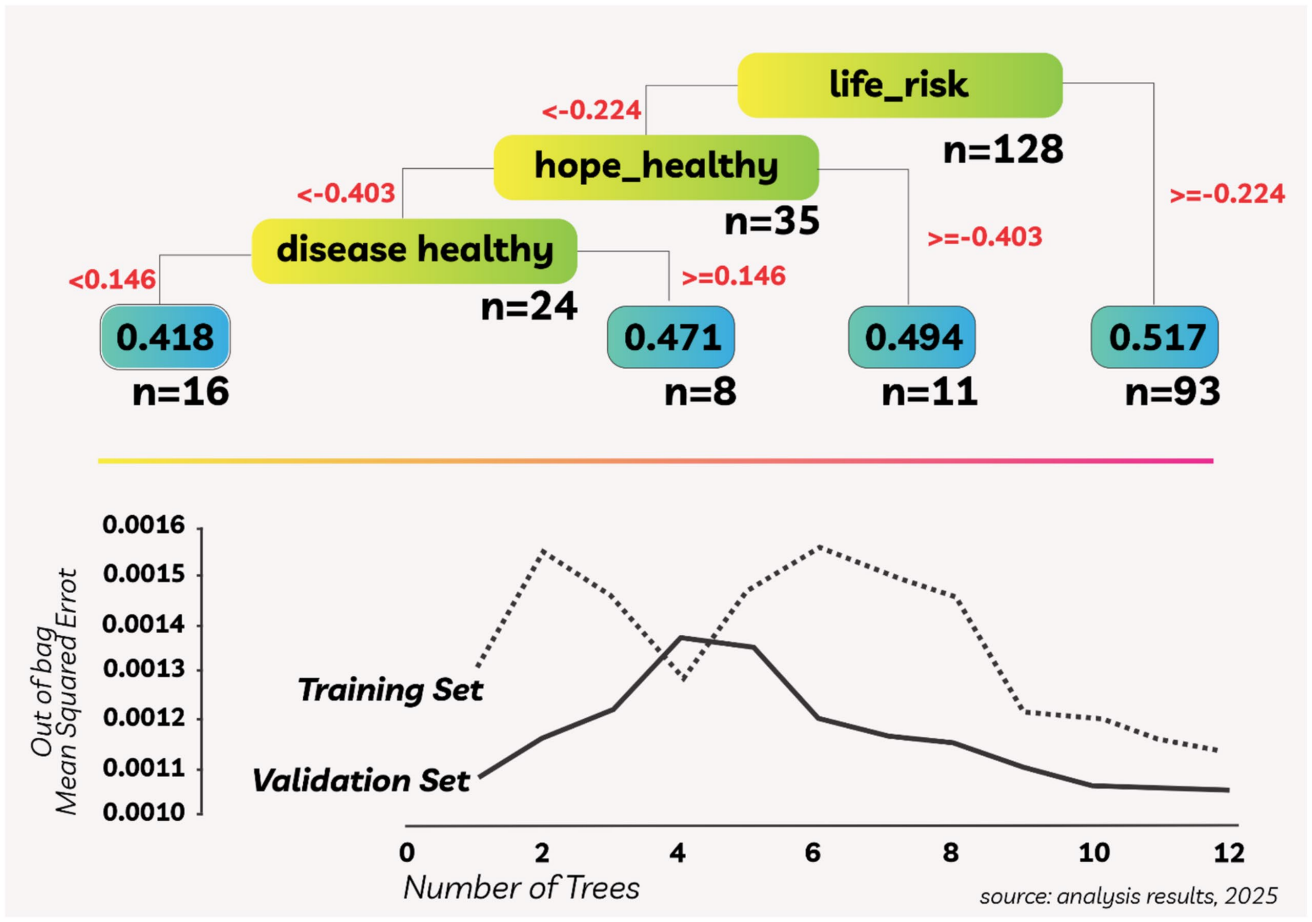
DTR on TAGANA health security determinants.

The results of statistical analysis conducted to understand the main factors affecting the health resilience of TAGANA disaster volunteers are visualized in Figure 1. Figure 1 shows the DTR structure that models the main factors of TAGANA health resilience, with life risk as the first separator at the threshold value of −0.224, followed by hope healthy (−0.403) and disease healthy (0.146). Each final node displays the average health resilience value and the number of individuals in the category, confirming that life risk is the most significant factor, supported by previous studies related to the impact of stress and high workload (30). Figure 1 shows the RFR curve showing that the decline in MSE occurred up to about five trees, the optimal point before the risk of overfitting increased. The RFR model has been shown to improve the accuracy of predicting the health resilience of disaster volunteers through the effective use of risk variables (31). This study emphasizes the importance of statistical and data-driven approaches in formulating policies to improve the health resilience of TAGANA members. Life risk is the dominant factor that reduces resilience, so risk mitigation through preparedness education, physical capacity building, and mental health interventions is needed. In contrast, hope and health help individuals cope with adversity, while training and fieldwork build resilience.

Policy implications emphasize a holistic approach based on three pillars: risk mitigation, strengthening psychosocial wellbeing, and optimization of field experiences through continuous training and hands-on learning.

## 4 Discussion

The study shows that an unhealthy lifestyle decreases health resilience, particularly in terms of physical and mental balance. The dominant factors were life risk (51.69%) and hope healthy (32.43%), while disease history (7.44%) and physical–mental balance (2.41%) contributed less, highlighting challenges in translating awareness into healthy behaviors. Unhealthy diets and lack of physical activity increase the risk of hypertension and metabolic disorders. Internationally, technology- and community-based health support programs—such as health monitoring apps in South Korea (32), social media modules in Australia (18), and online counseling in Germany and Italy (33)—provide models for intervention. Community-based approaches in Thailand and Mexico also promote local healthy lifestyles (34, 35). Compared with these initiatives, TAGANA operates with limited institutional health support, making hope healthy and community solidarity especially critical. This suggests that resilience may rely more on internal and cultural resources where systemic protections are weaker. This is consistent with recent evidence that affective temperaments significantly shape resilience under stress (36), while environmental exposures can further interact with biological pathways that influence vulnerability to mental health risks (37). Hope healthy may operate as a cognitive-emotional buffer, reducing the perceived severity of risks and facilitating adaptive coping (38). Likewise, social support enhances resilience through stress regulation, fostering collective efficacy and reducing burnout (39). By adopting such practices, TAGANA can develop holistic programs including technology, nutrition education, psychological support, physical activity, and mindfulness. Structured interventions such as stress management training and improved access to nutritious food are particularly needed.

Several international policies and practices can serve as valuable references for strengthening TAGANA’s capacity and health resilience. In New Zealand, ongoing preparedness and risk management training helps volunteers handle complex disasters effectively (40). Croatia implements a multilayer disaster response protocol that ensures coordinated action across health, logistics, and social sectors (41). In Latin America and Mexico, community-based programs like CERT empower local populations to strengthen grassroots health systems (35). Australia utilizes digital platforms to coordinate and integrate spontaneous volunteers during emergencies (42), while Malaysia emphasizes volunteer motivation—social, familial, and religious—as a basis for flood mitigation initiatives (43). Nepal has introduced a tourism volunteer framework that engages volunteers in rebuilding disaster-affected tourist destinations with integrated coordination (44). The United States ensures legal protections and cross-state licensing for healthcare workers participating in disaster response (45), and Iran has developed preparedness training based on experimental research to enhance volunteers’ long-term knowledge, skills, and attitudes (46). These global practices offer models that TAGANA can adapt for training, coordination, and protection. Yet structural gaps highlight the need to integrate psychosocial frameworks that build on cultural strengths such as collectivism and religious motivation.

This study also shows that dynamic field experience improves health resilience, reinforcing its policy relevance. TAGANA can adopt a wide range of global practices such as disaster education in schools and community engagement in Japan (47) and Taiwan (48), psychosocial support in Croatia (49), as well as emotion management in Sweden (50). CERT programs in Latin America are effective in strengthening local capacity (35), while adaptive training curricula in Iran improve volunteer skills (46).

Unlike contexts with strong legal and welfare protection, TAGANA volunteers remain unsupported by systematic safeguards. This underlines the urgency of aligning Indonesia’s system with the Sendai Framework for Disaster Risk Reduction (51), especially its priority to strengthen governance and protect frontline responders. International collaborations such as India’s disaster diplomacy can also expand TAGANA’s global role (52). These findings resonate with international evidence on the protective role of experience, but cultural values in Indonesia—such as collectivism, religious identity, and community solidarity—likely amplify this effect compared with more individualistic contexts. This underscores the need to contextualize global resilience frameworks to local socio-cultural realities rather than applying them uniformly. Further research should examine the influence of social support—family, community, and organization—on resilience, and test healthy lifestyle interventions such as structured exercise, balanced diets, and mindfulness. Longitudinal studies are needed to assess the long-term effects of life risk (e.g., PTSD, metabolic and emotional disorders) and explore protective mechanisms. Such work would bridge local empirical insights with global resilience agendas, ensuring TAGANA contributes not only to national disaster preparedness but also to international discourse on volunteer protection and health resilience. This research will support the design of real interventions by the government and non-governmental organizations to strengthen the physical and mental resilience of disaster volunteers in Indonesia. In addition, longitudinal studies are needed to track the cumulative effects of life risk exposure (e.g., PTSD, metabolic or emotional disorders) across time, while interventional trials can evaluate the effectiveness of structured programs—such as resilience training, peer-support groups, and lifestyle coaching—in strengthening volunteer health. This study has several limitations. Its cross-sectional design limits causal inference, and reliance on self-reported measures may introduce bias and fail to capture objective physiological resilience. Future studies should adopt longitudinal and mixed-method approaches to strengthen the validity and generalizability of findings.

## 5 Conclusion

This study shows that life risk (51.69%) and hope healthy (32.43%) are the main factors that affect the health resilience of TAGANA volunteers. Other factors—including field experience, disease history, and physical-mental balance—contributed less. These results emphasize the importance of integrating risk mitigation, psychosocial support, and healthy lifestyle education in TAGANA training. Policy recommendations include integrated training that combines technical skills and emotional resilience, periodic health monitoring, and cross-sectoral collaboration. However, this study has several limitations: the use of purposive sampling may reduce generalizability, the cross-sectional design does not capture long-term changes, and the reliance on self-reported Likert items may introduce response bias. Future research should adopt longitudinal and interventional designs to examine the cumulative effects of life risk, assess causal pathways, and evaluate the effectiveness of structured resilience programs. Cross-contextual comparisons across regions and disaster volunteer organizations would also provide broader insights into how cultural and institutional settings shape resilience mechanisms. Follow-up studies need to be longitudinal to evaluate the long-term impacts of risk exposure and changes in healthy behaviors. These findings can be the basis for strengthening the capacity of disaster volunteers in Indonesia and global practices.
